# The Effect of Cellulose Nanofibres on Dewatering during Wet-Forming and the Mechanical Properties of Thermoformed Specimens Made of Thermomechanical and Kraft Pulps

**DOI:** 10.3390/nano13182511

**Published:** 2023-09-07

**Authors:** Eirik Ulsaker Jacobsen, Simen Prang Følkner, Jørgen Blindheim, Dag Molteberg, Martin Steinert, Gary Chinga-Carrasco

**Affiliations:** 1 Department of Mechanical and Industrial Engineering, Norwegian University of Science and Technology (NTNU), Richard Birkelandsvei 2B, 7491 Trondheim, Norway; 2 Norske Skog Saugbrugs, Tistedalsgt. 9-11, 1772 Halden, Norway; 3 RISE PFI, Høgskoleringen 6b, 7491 Trondheim, Norway

**Keywords:** wet-forming, thermoforming, wood pulp, nanocellulose, food packaging, sustainable

## Abstract

Due to environmental concerns regarding single-use plastic materials, major efforts are being made to develop new material concepts based on biodegradable and renewable resources, e.g., wood pulp. In this study, we assessed two types of wood pulp fibres, i.e., thermomechanical pulp (TMP) and Kraft pulp fibres, and tested the performance of the fibres in wet-moulding and thermopressing trials. Kraft pulp fibres appeared to retain more water than TMP, increasing the dewatering time during wet-moulding and apparently increasing the compression resistance of the pulp during thermoforming. Additionally, cellulose nanofibres (CNF) were added to the pulps, which improved the mechanical properties of the final thermopressed specimens. However, the addition of CNF to the pulps (from 2 to 6%) had a further decrease in the dewatering efficiency in the wet-moulding process, and this effect was more pronounced in the Kraft pulp specimens. The mechanical performance of the thermoformed specimens was in the same range as the plastic materials that are conventionally used in food packaging, i.e., modulus 0.6–1.2 GPa, strength 49 MPa and elongation 6–9%. Finally, this study demonstrates the potential of wood pulps to form three-dimensional thermoformed products.

## 1. Introduction

Plastics have become an important part of modern society due to characteristics such as good mechanical properties, low weight and relatively low cost. However, there is increasing controversy regarding the environmental impacts of plastic, both in terms of fossil resources being used as raw material, and plastic litter in nature. In addition, biobased plastics (also classified as bioplastics) have appeared as an alternative to fossil-based plastic materials [1], although the production capacity is still limited and relatively expensive. Bioplastics may also have limitations regarding biodegradability, compostability and recyclability, and some bioplastics may contribute to marine littering [2].

Wood pulp is a renewable resource and has potential to replace some plastic products. Hence, the application of wood pulp fibres has expanded beyond the scope of conventional paper. There are various processes to obtain fibres from woody biomass, some of the most common are thermomechanical pulping (TMP), chemi-thermomechanical pulping (CTMP) and chemical pulping. These processes yield pulps with different chemical and structural characteristics suitable for different applications, e.g., packaging, tissue paper or printing paper.

The chemical Kraft process (also known as the sulphate process) is the most commonly used pulping process followed by TMP [3]. The TMP process uses high-temperature steam to soften the fibres before the mechanical refining, which yields a stronger pulp than purely mechanical refinement. Unfortunately, this comes at the cost of increased energy consumption during the production process. TMP fibre production requires more electricity, but fewer chemicals and fossil fuels [4]. In addition, the production yield of TMP fibres (>95%) is higher than that of Kraft pulp fibres (~50%) [5], which implies a reduction in pulp costs.

During the last decade, cellulose nanofibres (CNF), also called microfibrillated cellulose (MFC), have appeared as a new type of cellulose material that has been proposed as reinforcement of paper and plastic products [6,7]. The addition of CNF (4% concentration) to TMP handsheets demonstrated a significant effect on tensile index [8]. Taipale et al. [9] had the goal of assessing the mechanical properties and drainage times of Kraft pulps that were mixed with varying amounts of CNF. They found that there were a number of parameters that affected the drainage time of the pulp mixed with CNF such as pH, salt content or type of cationic polyelectrolyte. Furthermore, it was found that an increased CNF content did not only provide significant strength improvements to paper made from the pulp, but it also caused a significant increase in the drainage time [9]. Water drainage may also be affected by fibre morphology and chemical composition [10]. Additionally, the effect of CNF and primary fines was confirmed by Winter et al. [11], i.e., increasing the fraction of CNF and primary fines in Kraft pulps increased the dewatering resistance. Excessive drainage resistance is considered a major drawback as this may also lead to difficulties in industrial upscaling. It is expected that optimisation of the production processes could allow CNF to provide increased strength without compromising dewatering. An alternative approach was tested by Hii et al. [12] where the authors included mineral particles in a TMP furnish to improve drainability while applying CNF to compensate for the reduction in strength. The authors demonstrated that CNF contributed to internal bonding of fibres and mineral fillers and concluded that the approach could improve strength properties without affecting sheet pressability in the wet-end [12].

Pulp moulding is a technology area with major potential for wood pulp-based products. The earliest known patents for a method for making pulp products is dating back to 1890 [13] and the first machine for creating such products was patented in 1903 [14]. Pulp-based products have had a remarkable increase in popularity during the last years, especially due to increased environmental awareness. In recent years, environmental demands from both customers and governmental regulations have put pulp products in focus as a measure to eliminate the environmental footprint of single-use plastic materials. Their current use is mostly limited to packaging and various forms of food-related products, e.g., food trays. Moulded pulp and thermoforming of more complex three-dimensional objects is, however, still an area that requires development.

We hypothesized that CNF could improve the mechanical properties of thermoformed products. However, in order to be cost-efficient and thus industrially relevant, the potential mechanical strengthening of CNF should not compromise the dewatering resistance of the pulps during wet-forming. Therefore, the purpose of this study was to: (i) develop and demonstrate a set-up for making wet/thermoformed specimens for screening the mechanical performance of pulp furnish, (ii) perform a comparative study between TMP and Kraft pulp as raw materials for thermoformed products and (iii) reveal the effect of CNF on the strength and dewatering of thermopressed products.

## 2. Materials and Methods

### 2.1. Materials

Two pulps (bleached Kraft pulp and TMP) and a CNF sample (2.7% concentration, homogenized with 800 bar) were prepared by Norske Skog Saugbrugs (Halden, Norway). The TMP was produced by Saugbrugs from Norway spruce and the Kraft pulp was a commercial grade from Södra based on a Norway Spruce and Scotch Pine blend. The CNF was produced by Saugbrugs based on the same Kraft pulp.

### 2.2. Pulp Fibre Characterization

The characteristics of pulp fibres (length, width and fines) were determined with a FiberTester Plus device. The pulp fibres were diluted (0.1 g in 300 mL water) before the fibre measurements. The TMP and Kraft pulp were provided with a dry content of 30 and 90%, respectively. In order to increase the binding ability between fibres and the corresponding strength of the formed products, the Kraft pulp was refined in a PFI mill at 3500 revolutions previous to further processing.

The TMP fibres were prepared with bromine to stain lignin-rich areas on the surface of the fibres, as described by Reme et al. [15]. Kraft pulp fibres were prepared without staining for scanning electron microscopy (SEM) assessment. The images were acquired with a Hitachi SU3500 scanning electron microscope, in secondary electron imaging (SEI) and backscatter electron imaging (BEI) modes.

### 2.3. Sheets

Paper sheets (grammage 200 g/m^2^) of the two pulps were prepared for pressing trials. A sheet former (sheet dimensions of 19.5 cm × 19.5 cm) was used. Four sheets were manufactured for each pulp. The process included two distinct parts, i.e., the standardized sheet-making process in accordance with ISO 5269-1 [16] and the hot-pressing of the sheets in order to simulate the wet-moulding and thermoforming. The equipment applied for pressing was a Fontune Press LPB 300, with three adjustable parameters: temperature, pressure, and time. After preliminary testing, the following parameters were found to be optimal for pressing: temperature, time and pressure of 135 °C, 3 min and 60 kN, respectively. These parameters yielded dry and compact samples with good surface finishes. After being pressed the samples were stored and conditioned overnight in accordance with ISO 187 [17], i.e., temperature of 23 ± 1 °C and a relative humidity of 50 ± 2%.

Additional testing of the pulps and sheets of bleached Kraft pulp and TMP included addition of CNF at 2, 4 and 6 wt%. This was performed to verify the effect of CNF on; (i) dewatering of the pulp during forming and (ii) reinforcement of wet/thermoformed sheets. After mixing the pulps and CNF at the given concentrations the sheet-making process was performed according to ISO 5269-1 [16].

### 2.4. Thermoformed Test Specimens

**Figure 1:**
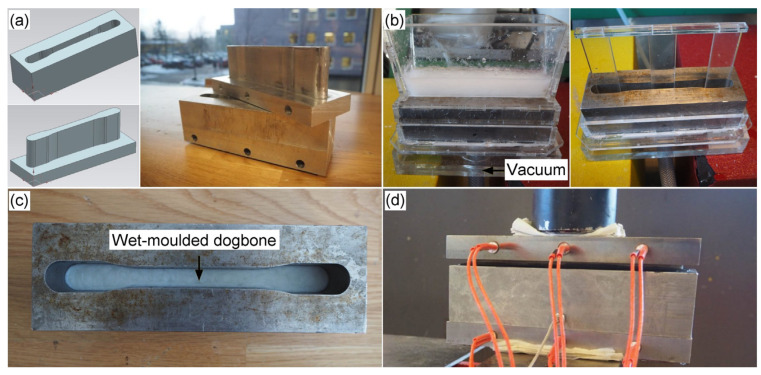
Set-up for wet- and thermoforming. (**a**) CAD designs of the negative and positive parts of the mould and the corresponding fabricated steel moulds. (**b**) Filtering and compression of the pulp. (**c**) Wet-moulded dogbone. (**d**) Thermopressing a dogbone sample.

The moulds and process used to make the dogbones are exemplified in Figure 1. Suspensions of the pulps were prepared (concentration 3%). The suspensions were poured into the dewatering box (Figure 1b) and the water was drained by applying vacuum, leaving wet-moulded dogbone samples in the mould (Figure 1c). The target thickness of the dogbones was 3.2 ± 0.4 mm. Preliminary testing revealed that 7.1 g TMP and 6.5 g Kraft pulp were necessary to produce dogbone samples with the correct sample thickness. The hot-pressing process consisted of first applying a force of 5 kN to the mould to compress the sample before the heating process started (Figure 1d). During the heating step, water started boiling, and the steam was allowed to escape while reducing the force to 0.2 kN to prevent the sample from expanding. After most of the initial water had evaporated (when no steam or water was observed) the force was increased to 1 kN for the last phase (Supporting Information, Figure S1). Five TMP and Kraft dogbone specimens were prepared. After moulding, the samples were conditioned in accordance with ISO 187 [17].

A similar approach was applied for thermoforming pulp fibre bowls (target thickness 3 mm), from the TMP and Kraft fibres. The pulps (80 g, concentration 2%) were dispersed (Lorentzen and Wettre pulp disintegrator, Kista, Sweden), posteriorly filtered through a 190 × 190 mm mould under vacuum and dewatered in a sheet press (PTI Austria GmbH, Laakirchen, Austria). The filtercake was then dried to a moisture content of approx. 20% before hot-pressing. The hot-pressing process was similar to the process applied for the dogbones and used 2 MPa (5 min), 0.08 MPa (4 min, release of evaporated moisture) and finally 0.4 MPa (10 min). The temperature during hot-pressing was 150 °C.

### 2.5. Mechanical Testing of Sheets and Dogbone Specimens

Tensile testing of the sheets was performed with a Lorentzen and Wettre Tensile Tester, using a constant rate of 100 mm/min. The length and width of the testing strips were 100 mm and 15 mm, respectively. Thickness was measured at five different points per sheet, using a micrometer, in accordance with ISO 534 [18]. Tensile testing of the dogbone samples was performed on MTS Criterion Model 42 tensile testing unit using a rate of 5 mm/min.

## 3. Results and Discussion

### 3.1. Fibre Characteristics

**Figure 2:**
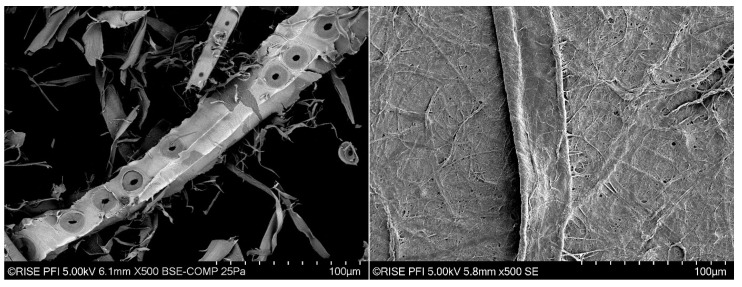
SEM analysis. (**Left**) SEM image of TMP acquired in BEI mode. Note the bright surface of the middle TMP fibre which corresponds to lignin-rich areas. (**Right**) A Kraft pulp fibre (middle area), surrounded by fibrillated material and nanofibers which form a relatively smooth surface.

Some morphological characteristics of fibres are provided in Figure 2 and Table 1. The two fibres differ significantly in morphology, being the TMP fibres shorter, wider and with a significantly larger fraction of fine materials (Table 1). This is typical for this type of fibres as TMP is based on mechanical refining where the fibres are exposed to mechanical stresses, which cause a larger splitting of the fibre wall (see, e.g., [19,20]). Kraft pulping yields more collapsed fibres, leading to denser sheet structures and thus higher tensile index [21]. Additionally, refining can cause higher water retention and a stronger fibre network due to the morphological changes in the fibre structure [21]. Kraft pulp fibres contain a relatively low fraction of fines, which may affect the strength of fibre networks [22].

### 3.2. Thermopressed Sheet Samples

**Figure 3:**
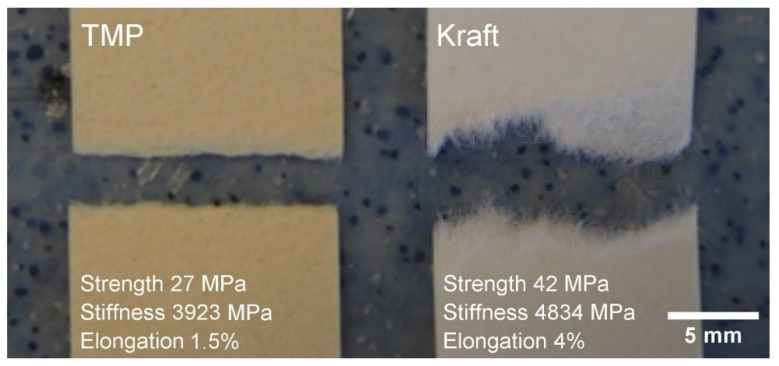
Examples of TMP and Kraft pulp samples and the corresponding mechanical properties.

The initial quantification of the mechanical properties of the sheets (200 g/m^2^) produced in this study revealed that the samples composed of Kraft pulp fibres had a considerably higher strength, stiffness and elongation at max strength (Figure 3 and Supplementary Information Figure S2). The relatively higher mechanical properties of the Kraft pulp fibres are due to the additional refining step performed to activate the fibres. A comparison between TMP and Kraft pulp samples shows that the force applied to the Kraft fibres was not only greatly exceeding that of the TMP but the elongation was considerably higher. On the other hand, the TMP and Kraft pulp fibres show similar elastic phases. Visual observation of the fracture areas revealed clear signs in line with the tensile test data, i.e., the TMP samples had a dense and brittle breaking point compared to the Kraft fibres which had a much more ductile behaviour with more fibres that seemed to have been intertwined before the break (Figure 3).

**Figure 4:**
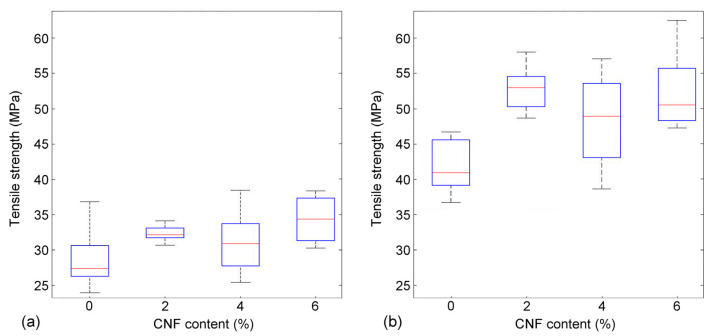
Tensile strength of TMP (**a**) and Kraft pulp samples (**b**).

The tensile strength of each series is presented in Figure 4. Although there are some variations between the TMP and Kraft samples, the results confirm that the CNF addition improves the strength of the material, especially in the case of the Kraft pulp samples (Figure 4b). The increase in strength is approx. 16% and 24% for the TMP and Kraft pulp, respectively, when adding 2% CNF. The CNF has been produced from the same Kraft pulp applied in this study. Hence, the larger increase in strength for the Kraft samples could be caused by a potentially better compatibility between CNF and the Kraft pulp fibres. On the other hand, TMP fibres have areas on the fibre surfaces covered by lignin (Figure 2, Left), which is less hydrophilic than cellulose and may limit the binding ability between the fibrous components. The increase in strength from the control sample (0% CNF) to the samples containing increased fractions of CNF (2, 4 and 6%) was consequent and as expected (see also [9]).

**Figure 5:**
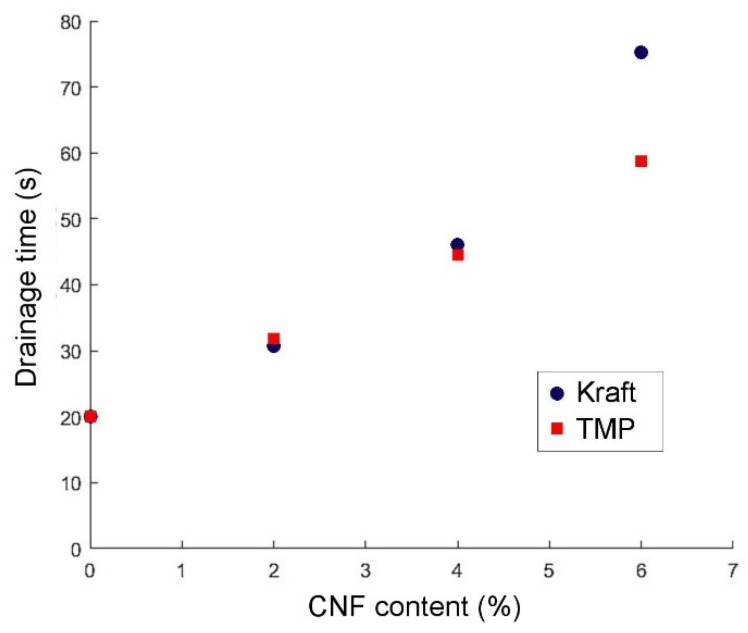
Drainage time of TMP and Kraft pulp samples (200 g/m^2^) as a function of CNF content.

The measured drainage time of the TMP and Kraft pulp samples as a function of CNF content showed a prolonged drainage time as the CNF content was increased (Figure 5). This was especially the case for the Kraft pulp, which reveals a major increase with 6% CNF content. This trend confirmed previous studies where paper sheets containing CNF or primary fines revealed a higher drainage time [9,11]. It is worth mentioning that the ability to dewater or drain the pulp is an important characteristic in paper making and pulp moulding since it directly affects the production efficiency of paper or of moulded products. In mass-production applications, it is paramount that the draining time is kept to a minimum in order to keep production times low, allowing for a more profitable process.

### 3.3. Assessment of Moulded Dogbone Test Specimens

Testing with relatively thick paper samples (grammage 200 g/m^2^) demonstrated that the drainage time increased linearly as the CNF content increased (Figure 5). At the same time, the addition of >2% CNF did not have a major contribution to the mechanical properties of the manufactured samples (Figure 4). Hence, we decided to further test 2% addition of CNF in moulded dogbone samples.

**Figure 6:**
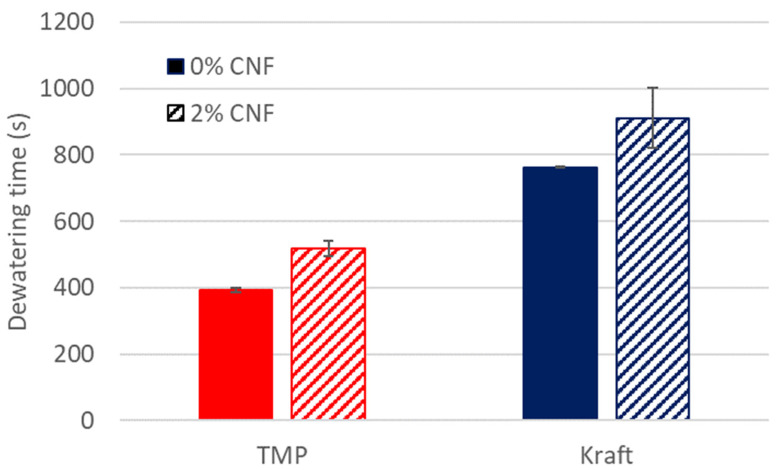
Dewatering time of wet-moulded dogbones of TMP and Kraft pulp, without and with addition of 2% CNF. The solid columns correspond to the TMP (red) and Kraft pulp (blue). The pattern columns correspond to the TMP (red pattern fill) and Kraft (blue pattern fill) with 2% CNF addition.

The results revealed that there was a clear tendency for the Kraft pulp to be more difficult to compress than the TMP. The TMP would usually be fully compressed within a minute of the initial force being applied (Supporting Information, Figure S1). However, the Kraft pulp required roughly five minutes of the compression phase before the targeted thickness was achieved. Considering that the Kraft samples required more time to dewater (dewatering time ~800 s) than the TMP samples, (dewatering time ~400 s) (Figure 6) this indicated that the Kraft samples most probably retained more water. This was also confirmed by visual inspection of the wet-formed samples. The greater retention of water and relatively excessive water content of the Kraft pulp fibres were probably the causes for the longer time required for hot-pressing to achieve the targeted thickness of the specimens.

The addition of 2% CNF caused a significant increase in the dewatering time during wet-forming of the dogbone specimens (Figure 6). It was thus expected that the prolonged dewatering time and potential increase in water retention in the Kraft pulp samples could become a challenge during the production of dogbone specimens. The moulded Kraft samples showed clear signs of not only smaller wet pockets, but a larger affected area that covered the entire narrow section of the dogbones specimens (Supporting Information, Figure S3). This effect seemed to be the result of water not being able to fully escape during drying. The samples had to air-dry after moulding to get rid of the excess moisture, which in turn created a wrinkled surface that further deteriorated the corresponding visual appearance.

The Kraft samples (with 2% CNF) also showed a tendency for the centre of the dogbone specimen to be thinner than the edges. This effect created a concave cross-section which was not desirable as it would make the calculation of mechanical properties more difficult. These negative effects could potentially have been alleviated with a more efficient filtering process; however, at this point, it was a challenge to add CNF to the Kraft pulp due to the increased probability of undesirable defects in the dogbone specimens. Although the strength of the moulded specimens could benefit from addition of minor amounts of CNF (Figure 4), the challenges due to dewatering may outweigh the potential benefits regarding the mechanical properties. Therefore, it was decided to manufacture dogbone specimens without CNF and perform the corresponding mechanical analysis. In addition, Negro et al. [23] demonstrated that the dry strength of thermopressed paper sheets was more affected by hot-pressing settings than by the addition of micro/nanofibrillated cellulose, indicating that CNF may not be necessary to improve the mechanical performance of thermopressed products.

**Figure 7:**
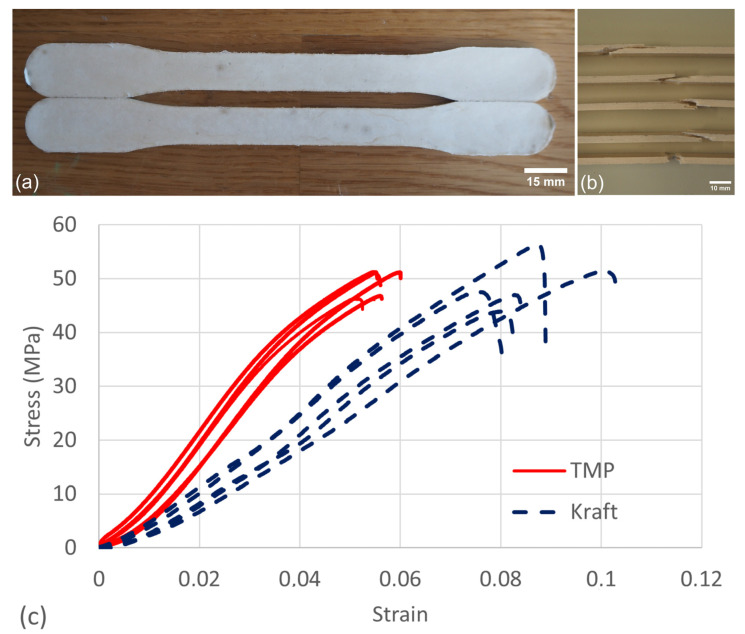
Mechanical testing of dogbones of TMP and Kraft pulp fibres. (**a**) Intact dogbone samples before testing. (**b**) Examples of the fracture area of tested dogbone samples. (**c**) Stress–strain curves for TMP and Kraft pulp fibres.

Figure 7 shows the stress–strain curves of the moulded dogbone samples. Note that the strength is similar in both samples. On the other hand, the strength of the moulded dogbones was considerably higher than that of the sheets. The TMP sheet broke in a more brittle manner than the Kraft samples (Figure 3). The sheets were relatively thin (thickness of 359–422 mm) compared to the thick dogbone samples (~3.2 mm). The higher strength of the dogbone samples is most probably due to the higher density of the thermoformed dogbones which was approx. 0.74 (Kraft) and 0.76 g/cm^3^ (TMP) compared to the sheet samples, approx. 0.47 (Kraft) and 0.56 g/cm^3^ (TMP).

In general, the moulded samples also revealed more linear stress–strain development than the paper sheets (Supplementary Information, Figure S2). Additionally, the stress–strain curves of the Kraft samples were more linear than the TMP samples, which revealed a more S-shaped curve. The characteristics of the stress–strain curves suggested that the thicker moulded samples had a much more elastic behaviour compared to the paper sheets, which showed a very standard elastic-plastic behaviour.

The Young’s modulus for the moulded samples was significantly lower (between 0.6 GPa for the Kraft sample and 1.2 GPa for the TMP sample), compared to the Young’s modulus of the paper sheets, which was between 4 and 5 GPa. The paper sheet curves were overlapping in the linear elastic region, with minor variation in modulus (Supplementary Information, Figure S2). The moulded samples, on the other hand, showed a greater degree of scattering. The difference in modulus suggested that the paper sheets were stiffer than the moulded samples and, thus, that moulded samples were more inclined to deform elastically at lower loads. The lower stiffness of the moulded dogbones may be due to the relatively mild pressing conditions applied in this study. By applying higher pressure during thermoforming, Ruwoldt and Tanase-Opedal [24] reported Young’s modulus values between 2.2 and 3.6 GPa for thermopressed CTMP specimens having densities between 0.95 and 1.2 g/m^3^. This observation exemplifies that the densities and mechanical properties can be tailored depending on the final product requirements.

The elongation of the Kraft dogbone specimens was still superior but not to the same extent as with the paper sheets. In addition to this, the strength of the TMP dogbone specimen was on the same level as that of the Kraft sample. This feature could allow for a future material to be made out of either TMP or Kraft depending on the mechanical requirements of specific applications.

One interesting aspect of this study was in comparing the moulded fibre samples to different types of plastic. The materials chosen for this comparison were six relevant plastics used for food packaging, including polyolefins (polyethylene terephthalate (PET), high- and low-density polyethylene (PE), polypropylene (PP)) and polyester (polylactic acid (PLA)) (Table 2). For some of these plastics, there were several possible production methods and structures that require different mechanical properties. Because of this, some of the data in Table 2 have a relatively large span, especially in terms of elongation. The comparison demonstrated that despite some differences, the modulus and strength of the moulded pulp were similar to the selected plastics. Although the elongation of PLA was similar to that of the pulp fibres, the elongation of the polyolefins was much greater. Elongation can be tailored by processing variables and drying, but relatively low elongation (<~10%) is typical for pulp fibre materials [25]. Despite the differences in elongation, the thermoformed samples comply with the mechanical requirements of some plastic materials that are applied for food packaging.

### 3.4. Demonstrators

**Figure 8:**
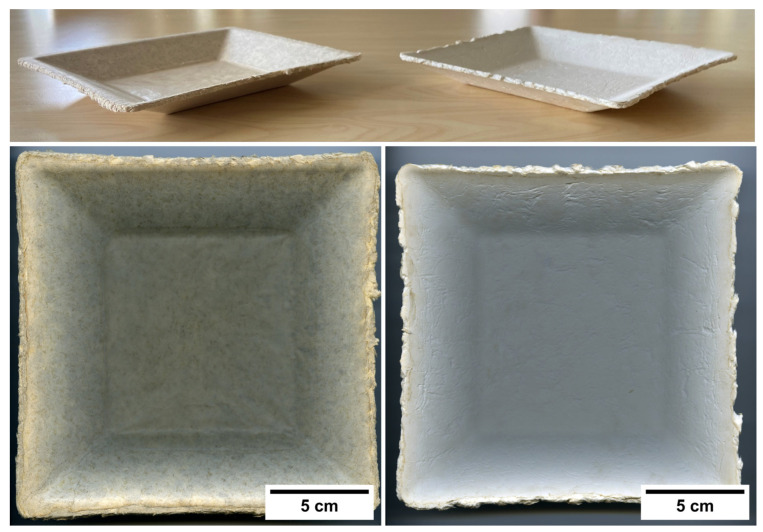
Thermoformed bowls of TMP (**left**) and Kraft pulps (**right**).

Taking into consideration that thermoformed pulp products can compete with some plastic food packaging, we demonstrated in this study the applicability of the pulp fibres for thermoforming food bowls (Figure 8). Note that in this study the Kraft pulp bowl has a rougher surface and higher degree of shrinkage compared to the TMP bowl. This behaviour is explained by the higher drying shrinkage known for bleached Kraft pulp fibres [28]. Although both types of demonstrators were solid and seemed to be adequate to be used in contact with relatively dry food, the TMP bowl had a smoother surface compared to the Kraft pulp bowl. Presently, the demonstrators do not have any surface modification to provide a liquid barrier. Some strategies have been proposed recently in this respect, including using lignin in the bulk to reduce hydrophilicity [24] or surface coatings containing CNF or combinations with chitosan [29,30,31]. However, such strategies only seem to reduce the liquid absorption. Hence, it still seems challenging to compete with the liquid-resistant PE layer applied on some pulp fibre products.

It is worth to keep in mind that the migration of substances, in the form of microplastics and nanoplastics, from packaging materials to food during storage or heating is known to occur for plastics, e.g., PP and PE [29]. Contrary to wood fibres that do not melt, the melting of PP and PE starts at approx. 50 °C, with peaks between roughly 110 °C and 165 °C [32]. Wood, fibres and paper in contact with food are regulated under Regulation (EC) No. 1935/2004 [33] and are considered safe for consumers. However, there may be other aspects that can have negative impact on health when using packaging based on these materials. For example, during the processing of paper or fibre-based materials, adhesives may be applied to improve strength and cohesion, and, as stated above, surfaces may be coated with polymers or waxes to provide a water barrier [34]. Wrona et al. [35] demonstrated how some dishes made of bamboo, palm leaf, wood and wheat pulp contained volatile compounds that exceeded the estimated daily intake limits set by the European Union. Due to the potential negative impact exceeding daily intake limits can have on human health, as well as nature upon exposure through littering, it would be beneficial to have fully bio-based packaging solutions as demonstrated in this study, which do not require adhesives to attain the required strength or barrier properties needed for certain food applications.

## 4. Conclusions

Fossil-based plastics and some bioplastics have limitations regarding their biodegradability, compostability and recyclability, and may also contribute to marine littering. Wood pulp fibres have the potential to replace some single-use plastic materials. Therefore, in this study we have made efforts to implement a setup for manufacturing dogbone specimens for screening the mechanical properties of wet/thermoformed pulp fibre specimens. The protocol implemented in this study contributes to rapidly characterizing wood pulp fibres in thermoforming trials. Two types of wood pulp fibres were assessed, i.e., TMP and Kraft pulp fibres. In addition, the pulps were complemented with CNF to improve the mechanical properties, which was demonstrated. Kraft pulp fibres appeared to retain more water, which increased the dewatering time during wet-moulding and apparently increased the resistance of the pulp to be compressed during thermoforming. Additionally, the increase in CNF from 2 to 6% had a further decrease in the dewatering efficiency in the wet-moulding process, and this effect was more pronounced in the Kraft pulp specimens. Based on these results, the manufacturing of thermoformed bowls made of pulp fibres without CNF was considered more efficient and demonstrated in this study.

## Figures and Tables

**Table 1:** FiberTester analysis of TMP, Kraft pulp fibres and CNF.

	Length (mm)	Width (mm)	Fibril Area (%)	Fibril Perimeter (%)	Fines (%)
TMP	1.46 ± 0.02	32.8 ± 0.0	10.4 ± 0.6	33.3 ± 1.3	42.5 ± 0.0
Kraft	2.08 ± 0.02	29.0 ± 0.1	2.1 ± 0.2	7.2 ± 0.3	25 ± 1
CNF	0.70 ± 0.02	25.7 ± 0.5	23.0 ± 0.0	56.8 ± 0.5	61.3 ± 0

**Table 2:** Mechanical properties of plastics and of the Kraft and TMP thermoformed samples produced in this study. The mechanical properties and densities of the selected plastic materials were obtained from [26,27].

Pulp Fibres	Young’s Modulus (GPa)	Tensile Strength (MPa)	Elongation (%)	Density (g/cm^3^)
TMP	1.2 ± 0.04	49.3 ± 2.5	5.6 ± 0.3	0.76
Kraft	0.6 ± 0.06	49.2 ± 4.8	8.6 ± 0.9	0.74
**Plastic materials**				
PET	2.95	55	100	1.38
HDPE	1.0	26	590	0.94–0.97
LDPE	0.3	10	625	0.91–0.94
PP	1.3	34	450	0.90–0.92
PLA	3.6	60	6	1.24

## Data Availability

Data will be made available on request.

## Supplementary Materials

The following supporting information can be downloaded at: https://www.mdpi.com/article/10.3390/nano13182511/s1, Figure S1: Force and temperature profile applied during the moulding process.; Figure S2: Stress–strain curves of thermopressed sheet samples; Figure S3: Examples of defects in dogbone samples.
