# Supplementary material for: The Effect of Cellulose Nanofibres on Dewatering during Wet-Forming and the Mechanical Properties of Thermoformed Specimens Made of Thermomechanical and Kraft Pulps
Source: Nanomaterials (Basel). 2023 Sep 7;13(18):2511. doi: 10.3390/nano13182511 (PMC10536136; doi:10.3390/nano13182511)
Supplement: Supplementary file 1 [file nanomaterials-13-02511-s001.zip › nanomaterials-2588246-supplementary.pdf]

nanomaterials

Supplementary information

The effect of cellulose nanofibres on dewatering during wet-forming and the mechanical properties of thermoformed specimens made of thermomechanical and Kraft pulps

Eirik Ulsaker Jacobsen[1], Simen Prang Følkner[2], Jørgen Blindheim[1], Dag Molteberg[2], Martin Steinert[1], Gary Chinga-Carrasco[3*]

[1] Department of Mechanical and Industrial Engineering, NTNU, Richard Birkelandsvei 2B, 7491 Trondheim, Norway
[2] Norske Skog Saugbrugs, Tistedalsgt. 9-11, 1772 Halden, Norway
[3] RISE PFI, Høgskoleringen 6b, 7491 Trondheim, Norway
* Correspondence: gary.chinga.carrasco@rise-pfi.no

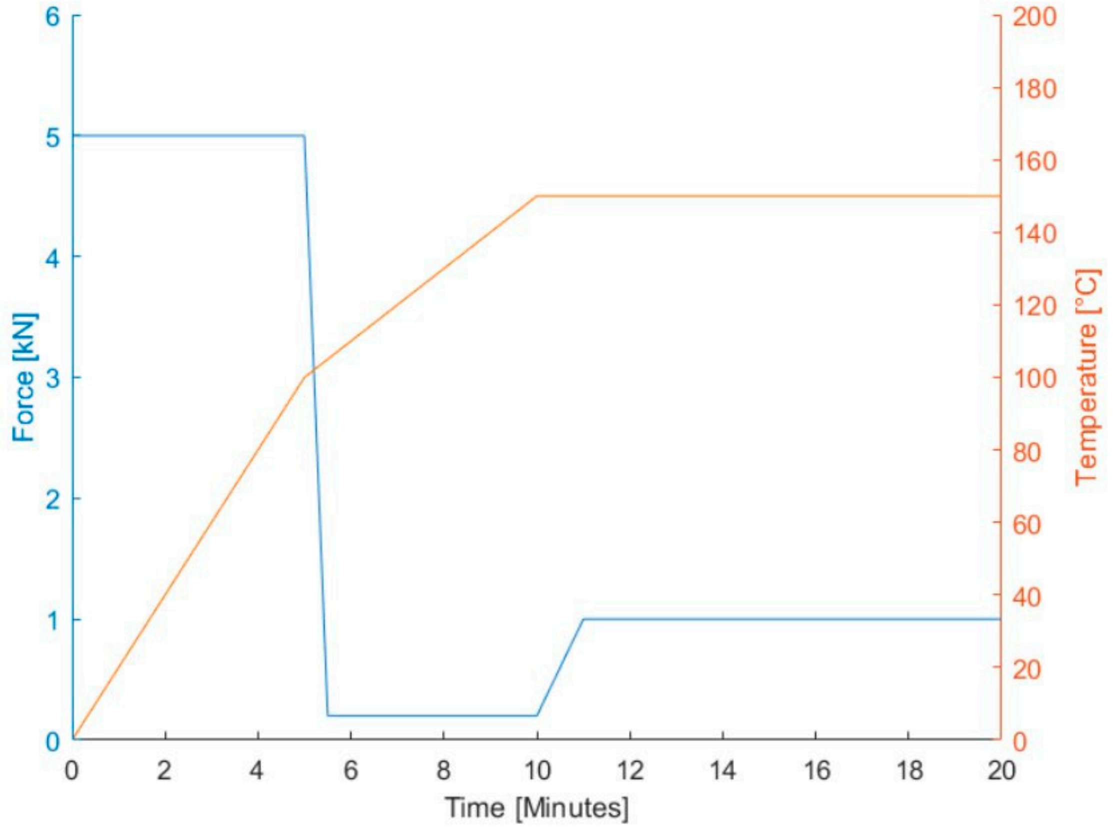

Figure S1 Force and temperature profile applied during the moulding process.

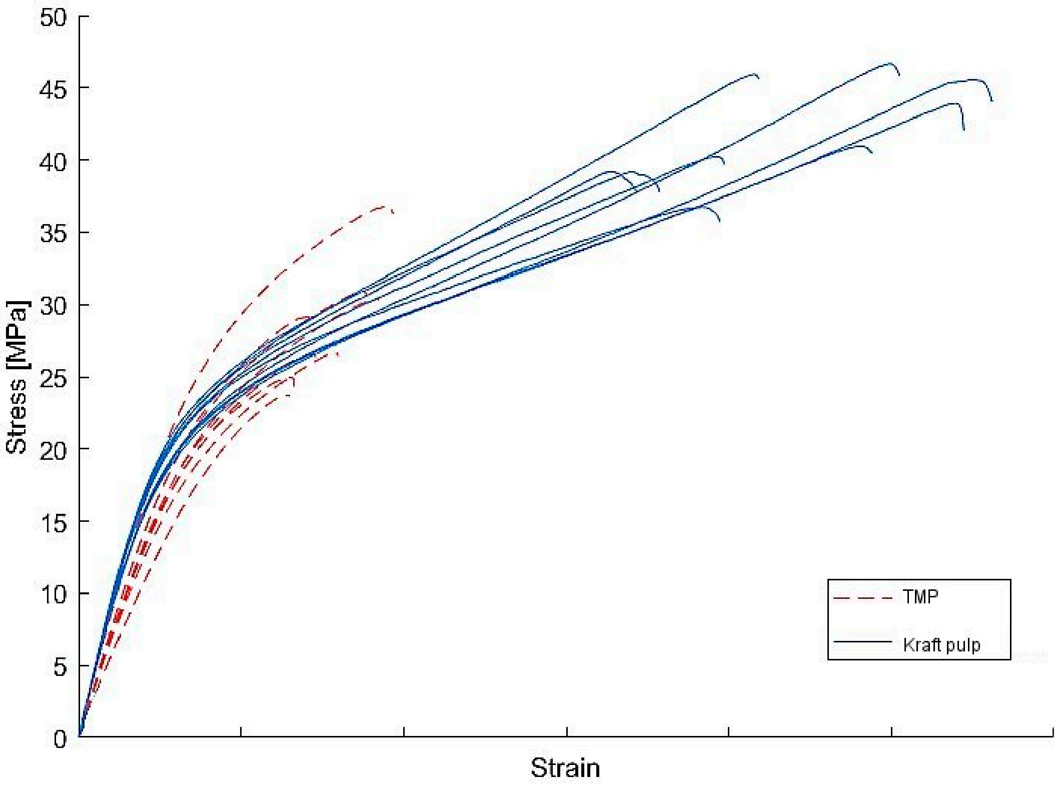

Figure S2 Stress-Strain curves of thermopressed sheet samples.

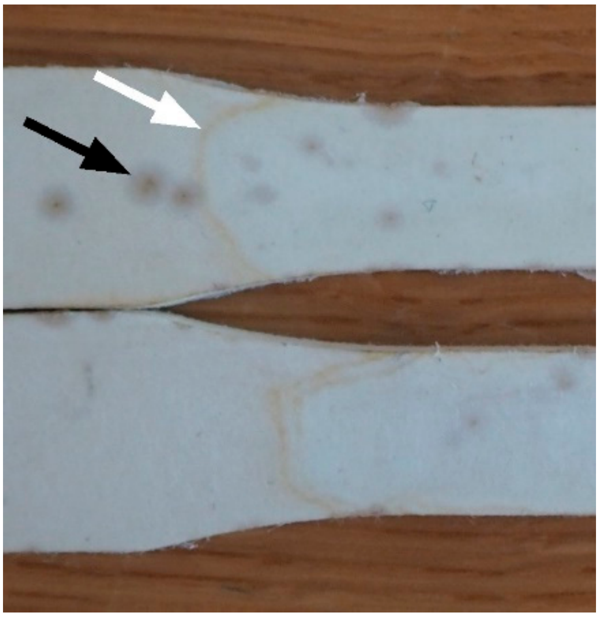

Figure S3 Examples of defects in dogbone samples. The border caused by water entrapment (white arrow) and spots (black arrow) are exemplified.

.
